# m4C DNA methylation regulates biosynthesis of daptomycin in *Streptomyces roseosporus* L30

**DOI:** 10.1016/j.synbio.2022.06.001

**Published:** 2022-06-17

**Authors:** Jiao-Le Fang, Wen-Li Gao, Wei-Feng Xu, Zhong-Yuan Lyu, Lie Ma, Shuai Luo, Xin-Ai Chen, Xu-Ming Mao, Yong-Quan Li

**Affiliations:** a Institute of Pharmaceutical Biotechnology, Zhejiang University School of Medicine, Hangzhou, 310058, PR China; b Zhejiang Provincial Key Laboratory for Microbial Biochemistry and Metabolic Engineering, 310058, Hangzhou, PR China

**Keywords:** N4-methylcytosine, DNA methyltransferase, Daptomycin, Transcriptional regulator, Secondary metabolism

## Abstract

Despite numerous studies on transcriptional level regulation by single genes in drug producing *Actinomyces*, the global regulation based on epigenetic modification is not well explored. N4-methylcytosine (m4C), an abundant epigenetic marker in *Actinomycetes*’ genome, but its regulatory mechanism remains unclear. In this study, we identify a m4C methyltransferase (SroLm3) in *Streptomyces roseosporus* L30 and multi-omics studies were performed and revealed SroLm3 as a global regulator of secondary metabolism. Notably, three BGCs in Δ*sroLm3* strain exhibited decreased expression compared to wild type. In-frame deletion of *sroLm3* in *S.roseosporus* L30 further revealed its role in enhancing daptomycin production. In summary, we characterized a m4C methyltransferase, revealed the function of m4C in secondary metabolism regulation and biosynthesis of red pigment, and mapped a series of novel regulators for daptomycin biosynthesis dominated by m4C methylation. Our research further indicated that m4C DNA methylation may contribute to a metabolic switch from primary to secondary metabolism in *Actinomyces.*

## 1 Introduction

Researchers have made earnest effort on transcriptional regulatory mechanisms in *Actinomycetes*, the main industrial producer of natural drugs, but little exploration has been devoted to epigenetic regulation [1,2]. DNA methylation is a ubiquitous epigenetic modification distributing from prokaryotes to eukaryotes [3]. Researchers have identified three different forms of DNA methylation in bacterial genomes: m6A (N6-methyladenine), m4C (N4-methylcytosine) and m5C (5-methylcytosine) [4]. While m5C is the predominant form in eukaryotes, m6A is the most prevalent form in bacteria [5]. m4C rarely exists in eukaryotes but is common in bacteria and archaea [6].

Methylation on DNA bases can change the activity of a DNA segment without changing the sequence [7]. Since the introduction and application of single-molecule real-time (SMRT) sequencing technology, we now have an efficient and potent tool to investigate the mysteries hidden in DNA's double helix [8]. Hence, “orphan” or “solitary” MTase, which lacks an accompanied restriction endonuclease, appeared to have an important function in gene regulation, such as Dam (DNA adenine methyltransferase) in *E. coli* and CcrM (cell cycle-regulated methyltransferase) in *Caulobacter crescentus* [9]. Previous studies in bacteria have revealed the importance of DNA methylation in DNA repair, gene expression, toxicity, and cell-cycle regulation [10,11], but the global regulatory functions of m4C modification in microorganisms remain unclear since this kind of methylation is not the major modification in bacteria [12]. In recent years, researchers have investigated the roles of m4C in epigenetic regulation, virulence, and transcription, but those are only the tip of a substantial iceberg [13,14].

*Streptomyces roseosporus* L30 is an industrial strain producing daptomycin, a cyclic lipopeptide antibiotic against Gram-positive pathogens [15]. Methylome analysis revealed the existence of m4C and m6A in the genome. A conserved motif, 5′-CGACNNNCTCC-3’/5′-GGAGNNNGTCG-3’ (methylated bases are underlined), showed nearly complete methylation of the adenine in the whole genome during exponential and stationary phase. Conversely, m4C exhibited a significant different distribution during two phases, suggesting its function in secondary metabolic regulation. So far, no physiological role has been assigned to irregular distributed m4C in bacteria. In this study, we identified three DNA methyltransferases, *sroLm1*-*sroLm3*, in the genome of *S. roseosporus* L30. And we identified SroLm3 as a m4C MTase and explored its function in regulation of daptomycin and other secondary metabolites production in *S. roseosporus* L30. Combining methylome analysis and transcriptome analysis, we found four unreported regulators involved in daptomycin biosynthesis, and several unreported regulators involved in the red pigment biosynthesis. Moreover, Orf4820 is a transcriptional repressor of daptomycin and red pigment production [16] and the generation of spores. Our study investigated the function of m4C modification in the regulation of secondary metabolism in *S. roseosporus* and indicated that DNA methylation plays a vital role in “metabolic switch” in *Streptomycetes.*

## 2 Material and methods

### 2.1 Strains, plasmids, and media

All strains and plasmids used in this study were listed in Table 1 and Table S1. *S. roseosporus* L30 is a main industrial strain to produce daptomycin [17]. Other engineered mutant strains were listed in Table 1. *Escherichia coli* TG1 (Novagen) and DH5α (Tsingke) were used as general cloning hosts for plasmids. *E. coli* ET12567/pUZ8002 [18] was used to introduce plasmids into *S. roseosporus* L30.

**Table 1:** Strains used and constructed in this study.

Strains	Description	Reference
***S. roseosporus***		
L30	Industrial strain used for production of daptomycin	[17]
L31	In-frame deletion of *sroLm1* in L30	This study
L32	In-frame deletion of *sroLm2* in L30	This study
L33	In-frame deletion of *sroLm3* in L30	This study
L30-*Δpks20*	In-frame deletion of core PKS of cluster 20 in L30	This study
L33-*Δpks20*	In-frame deletion of core PKS of cluster 20 in L33	This study
L33-oe-*orf1070*	L33 with overexpression of *orf1070*	This study
L33-oe-*orf2061*	L33 with overexpression of *orf2061*	This study
L33-oe-*orf4820*	L33 with overexpression of *orf4820*	This study
L33-oe-*orf4996*	L33 with overexpression of *orf4996*	This study
L33-oe-*orf5980*	L33 with overexpression of *orf5980*	This study
L33-*Δorf4008*	In-frame deletion of *orf4008* in L33	This study
L33-*Δorf4141*	In-frame deletion of *orf4141* in L33	This study
L33-*Δorf4759*	In-frame deletion of *orf4759* in L33	This study
L33-*Δorf5274*	In-frame deletion of *orf5274* in L33	This study
L33-*Δorf4820*	In-frame deletion of *orf4820* in L33	This study
L33-*Δorf5980*	In-frame deletion of *orf5980* in L33	This study

***E. coli***		
TG1	Cloning host	Laboratory
DH5α	Cloning host	Novagen
BL21 (DE3)	Expression host for regulatory genes	Laboratory
ET12567/pUZ8002	Conjugation host	Laboratory

An integrative plasmid pIJ8661 containing strong promoter ermEp*, was used for overexpression of genes [19]. The plasmid pKC1139 was used for in-frame deletion of genes [20]. The plasmid pTA2 (Toyobo) was used for amplification of inserted fragments and sequencing. Other engineered plasmids are also listed in Table S1.

LB liquid medium and LB solid medium were used for culturing of *E. coli.* The R5 solid medium was used for generation and sporulation of *S. roseosporus* L30 and the mutants. The MS solid medium was used for conjugation for in-frame deletion of DNA methyltransferases. The TSB liquid medium was used as seed medium and the YEME liquid medium was used as fermentation medium for daptomycin production. 1 mM sodium decanoate was added in YEME liquid medium every 12 h starting from 60 h.

Four solid media: YMG, ISP4, R5 and MM, were used for phenotypical characterization of *S. roseosporus* at 30 °C. Four liquid media: YMG, MM and R5, YEME (with or without feeding of sodium decanoate) were used to detect the metabolic profiles in fermentation experiments.

### 2.2 Plasmid construction

The primers used in this study are listed in Table S2. The linearized vectors were generated by digestion of selected restriction endonucleases (Thermo Scientific) and the fragments were amplified by KOD Plus Neo (Toyobo). Amplified fragments were inserted into linearized vectors via ClonExpress MultiS One Step Cloning Kit (Vazyme) and transformed into DH5α for further study.

### 2.3 Construction of mutants of *S. roseosporus* strains

Integrative plasmids of pKC1139 containing homologous arms of the selected genes were cloned into *E. coli* ET12567/pUZ8002 separately. Then the plasmids were transformed into *S. roseosporus* via conjugation. Then the gene knock-out mutants were obtained by using the strategy of in-frame deletion and identified by PCR with primer pairs listed in Table S1.

For overexpression, genes were cloned and integrated into plasmid pIJ8661 via ClonExpress II One Step Cloning Kit. Engineered plasmids were transformed into *S. roseosporus* via conjugation and then integrated into the genome. The mutants were identified by PCR with primers listed in Table S1.

### 2.4 Morphologic observation of mutants

Solid media used for morphologic observation were mentioned above. All generated mutants were cultured on R5 solid medium for 2 rounds. Spores were collected and streaked on selected solid media. Petri dishes were kept in sealed plastic bags and placed in bacteriological incubator at a temperature of 30 °C for several days. The growth status was recorded every two days.

### 2.5 Fermentation and metabolic analysis of secondary metabolites of generated mutants

All generated mutants were cultured on R5 solid medium for 6–7 days. Spores were collected and inoculated into flasks with 35 mL TSB liquid medium and shaken at 250 rpm, 30 °C for about 36 h. 1.4 mL of the culture was inoculated into selected fermentation medium at an OD 0.4 and shaken at 250 rpm, 30 °C for about 7 days. The samples were treated with equal volume of methanol and centrifuged. The supernatants were collected and filtered through a Millipore membrane for HPLC analysis. The secondary metabolites were analyzed by 1260 Infinity II LC System (Agilent Technologies) using the method described in Table S3A.

### 2.6 Fermentation and HPLC analysis of daptomycin and A21978C_1-3_

Spores of *S. roseosporus* prepared from R5 solid medium were inoculated into TSB liquid medium. The cultures were grown at 30 °C on a rotary shaker at 250 rpm for about 30 h as the seed culture. 1.4 mL of seed culture was inoculated into flasks containing 35 mL of YEME liquid medium and then fermented at 30 °C on a rotary shaker at 250 rpm for 6 days. 1 mM sodium decanoate acid was added every 12 h starting at 60 h. 500 μL of the fermentation broth was sampled every 12 h starting at 72 h. The samples were treated with equal volume of methanol and centrifuged. The supernatants were collected and filtered through a Millipore membrane for HPLC analysis.

Daptomycin and A21978C_1-3_ were analyzed by HPLC using the method described in Table S3B.

### 2.7 Prediction of biosynthetic gene clusters of secondary metabolites

Prediction of biosynthetic gene clusters (BGCs) of secondary metabolites was done using antiSMASH 6.0 (https://antismash.secondarymetabolites.org/) [21].

### 2.8 SMRT sequencing

Shaking flask fermentation experiments in YEME liquid medium of *S. roseosporus* were done as mentioned above. 1 mL of the fermentation broth was harvested at different time points. Samples were centrifuged, and the supernatant was removed. High-quality genomic DNA was extracted using a modified CTAB method. Qualified genomic DNA was fragmented using G-tubes (Covaris) and then end-repaired to prepare SMRTbell DNA template libraries (with a fragment size >10 kb) according to the manufacturer's instructions (Pacific Biosciences). Library quality was analyzed by Qubit, and average fragment size was estimated using an Agilent 2100 Bioanalyzer (Agilent Technologies). SMRT sequencing was performed using a Pacific Biosciences Sequel II sequencer and standard protocols (Benagen) using Sequel II Binding Kit 2.0 chemistry.

PacBio library was constructed containing fragments at ∼10 kb, and whole genome sequencing was performed with the PacBio Sequel II system. The PacBio reads were *de novo* assembled using the Microbial Assembly (SMRTLink v8.0), HGAP4 and Canu (v1.6) software.

### 2.9 RNA-seq analysis

Hyphae were collected as mentioned in SMRT sequencing. Total RNA was extracted, and purity was checked using the NanoPhotometer spectrophotometer (IMPLEN). 3 μg RNA per sample was used as input material for the RNA sample preparations. Sequencing libraries were generated using UltraTM RNA Library Prep Kit for Illumina (NEB) following manufacturer's recommendations and index codes were added to attribute sequences to each sample. The clustering of the index-coded samples was performed on cBot Cluster Generation System using TruSeq PE Cluster Kit v3-cBot-HS (Illumina) according to the manufacturer's instructions. After cluster generation, the library preparations were sequenced on an Illumina Hiseq 4000 platform and 125 bp/150 bp paired-end reads were generated. Clean reads were aligned to the reference genome using Hisat2 v2.0.5.

FPKM of each gene was calculated based on the length of the gene and reads count mapped to this gene. Differential expression analysis was performed using the DESeq2 R package (v1.16.1). Genes with an adjusted *P*-value <0.05 found by DESeq2 were assigned as differentially expressed.

### 2.10 EMSA assay

Regulatory genes were cloned and integrated into linearized pET28a and transferred into *E.coli* BL21 (DE3). 1 mM IPTG was used to induce the protein expression after the cells reach mid-log growth (OD_600_ = 0.5–0.6). Proteins were purified with Ni-NTA resin (Novagen) and purity was confirmed by SDS-PAGE.

Promoter regions were cloned and integrated into a plasmid. 5′-FAM-labeled primer was used to obtain DNA probes for EMSA assay. Each binding reaction system contained 100 ng of probes and an increasing amount of protein. The results were confirmed by native-PAGE and visualized by ImageQuant LAS4000mini (GE).

## 3 Results

### 3.1 m4C DNA modification is involved in the regulation of secondary metabolism in *S. roseosporus* L30

The industrial fermentation cycle can be 132 h or longer. A rapid growth induced by polar growth of the hyphae in exponential phase occurs in first two days [22]. At the end of exponential phase, a brief transition phase precedes stationary phase. In *Streptomyces griseus*, the hyphae grow rapidly until around 48 h, the stationary phase will last for several days depending on the growth condition in the medium [23,24]. We gathered the hyphae at 24 h and 72 h in YEME liquid medium to represent cells in exponential growth phase and stationary phase to analyze the difference in methylome between primary and secondary metabolism. Then we processed SMRT sequencing with gDNA of *Streptomyces roseosporus* L30 (Table S4). SMRT-seq identified two types of DNA modifications, m4C and m6A (Fig. S1b) at both time points and m6A modification mostly occurred in a reverse complementary sequence 5′-CGACNNNCTCC-3’/5′-GGAGNNNGTCG-3’. We checked REBASE database but found no previous study reporting this recognition sequence, so this is a unique motif modified by an unrecognized DNA methyltransferase. Furthermore, almost all conserved motifs at two time points were fully modified.

Apart from the conserved modified motif, we also found randomly distributed methylated DNA bases, including m4C and m6A, spreading across the whole genome of *S. roseosporus* L30 (Fig. S1). In general, there were 6279 (24 h) and 6788 (72 h) random m4C in total, while we found 939 cytosines were modified at both points. The motif prediction provided no conserved motif in modified areas of m4C, but we counted all bases within a range of 20 bases adjacent to the m4C modified bases and found a priority of GCGG motif at both time points (Fig. S1d). We can conclude that the methylome across the whole genome changes during the fermentation period and that the distribution of m4C could be connected to secondary metabolism.

### 3.2 In-frame deletion of *sroLm3* exhibited a notable alteration of secondary metabolism in *S.roseosporus* L30

**Fig. 1:**
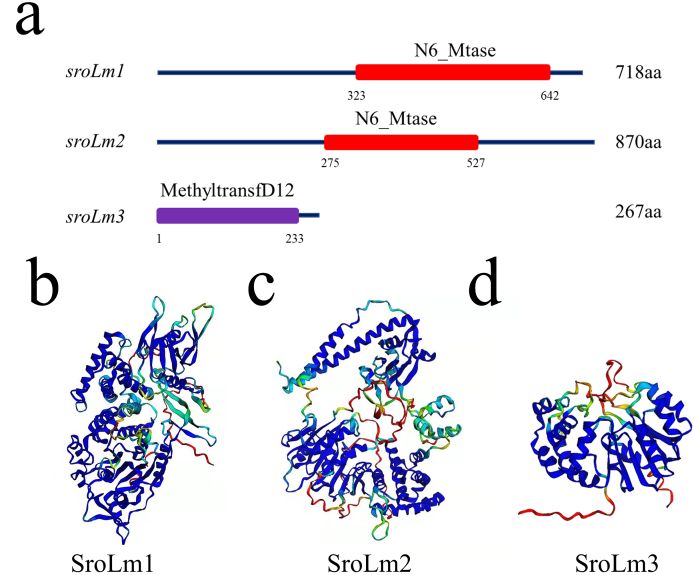
DNA methyltransferases in *S.roseosporus* L30. (a) conserved motifs in predicted DNA methyltransferases provided by NCBI. (b–d) 3D structures of predicted DNA methyltransferases characterized in *S.roseosporus* L30 predicted by AlphaFold2 provided by Google.

Genome mining of DNA methyltransferases by NCBI BLAST and REBASE database [25] online revealed three candidates named SroLm1-SroLm3 (Table 2) in *S. roseosporus* L30 (Fig. 1a). We constructed 3D models of the three candidates via AlphaFold2 [26] provided by Google and all three DNA methyltransferases were predicted as m6A DNA methyltransferases (Fig. 1b). Moreover, SroLm1 was predicted to be responsible for the methylation of the unique motif 5′-CGACNNNCTCC-3’/5′-GGAGNNNGTCG-3′ because SroLm1 has a co-transcriptional recognition protein. All three predicted DNA MTases were “orphan” MTase without coupled restriction enzymes.

**Table 2:** Genome-mining of candidates of DNA MTase in *S.roseosporus* L30.

DNA MTases	Location	Type/subtype	length (aa)
*sroLm1*	Chromosome	II gamma	718
*sroLm2*	Chromosome	II gamma	838
*sroLm3*	Chromosome	II alpha	303

**Fig. 2:**
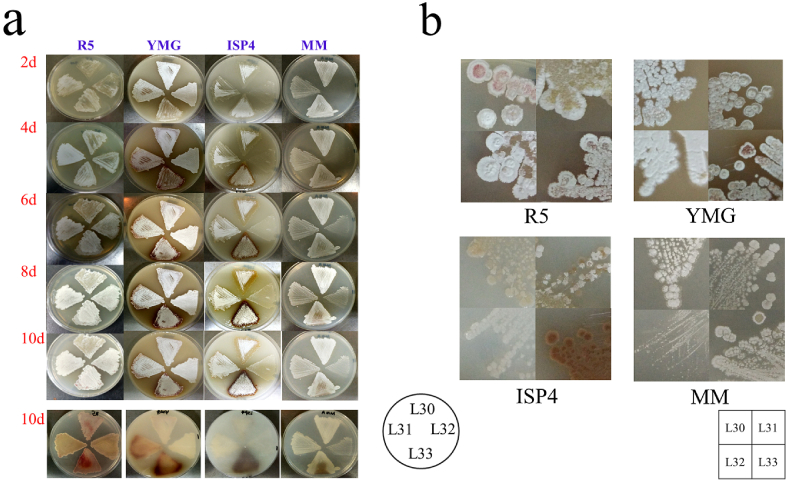
Morphological diversities on different solid medium in WT and three mutants. (a) Growth status of WT and three mutants on different solid media. L33 produced a brown pigment on ISP4 and MM solid medium. (b) Colony morphology exhibited on different solid media of WT and three mutants at 10d.

Three candidates *sroLm1-sroLm3* were knocked out respectively by homologous double-crossover to generate mutant strain L31-L33 (Fig. S2). Since DNA methylation is a ubiquitous epigenetic mark, and previous researchers demonstrated this modification can globally influence the phenotype [27], four kinds of solid medium, R5, YMG, MM and ISP4, were used for phenotypical observation. Three mutants and L30 strain were cultured in those solid media for up to 10 days to identify their functions in morphological development. We captured the images of the growth status of the mutants and L30 strains every two days (Fig. 2a).

Notably, L31 exhibited almost no morphological difference on all four kinds of solid medium compared to L30. L32 exhibits significant growth retardation on ISP4 and MM solid medium and we also observed smaller colonies on MM solid medium compared to other strains (Fig. 2b). Due to the single nitrogen source used in ISP4 ((NH_4_)_2_SO_2_) and MM (l-asparagine) solid medium, we believe that SroLm2 plays a vital role in nitrogen utilization.

**Fig. 3:**
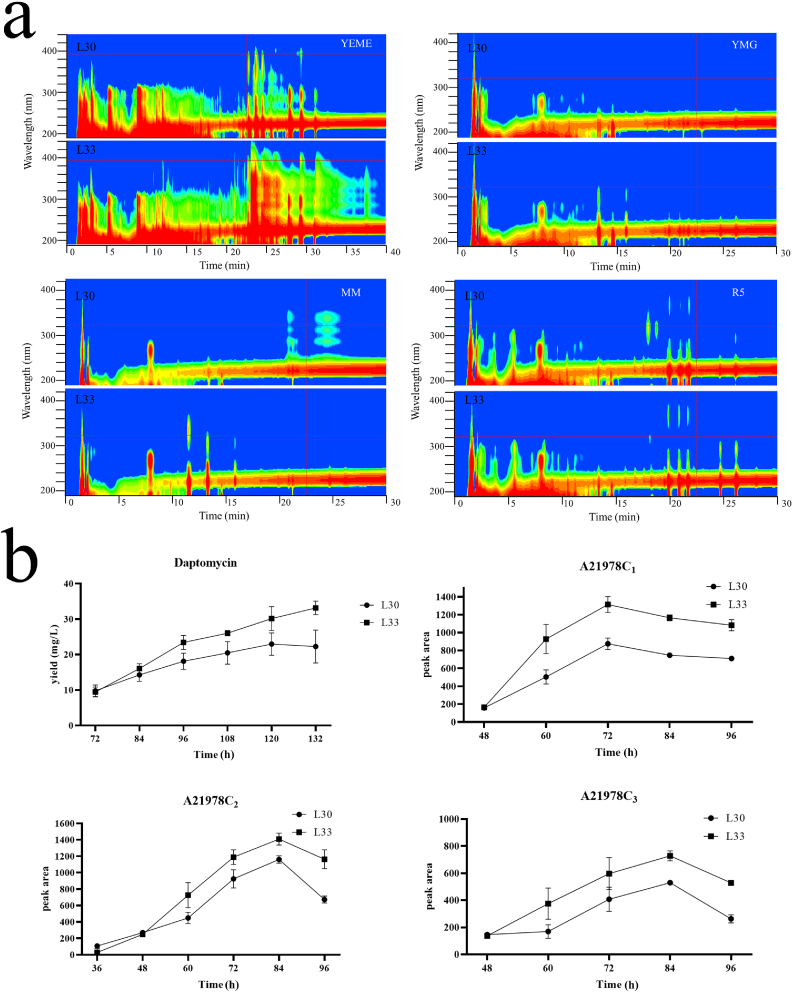
HPLC analysis with metabolic profile of fermentation broth in four kinds of liquid medium between L30 and L33. (a) Mutant L33 revealed different Metabolic profiles in all four different liquid media (YEME/YMG/MM/R5 liquid medium). (b) Yield of daptomycin and its analogs A21978C_1-3_ of L30 and L33 in fermentation experiments (*n* = 3, mean with SD).

Mutant L33 produced more pigments cultured on all four media. Particularly, a deep brown pigment was observed when cultured on ISP4 and MM solid medium. A fermentation experiment in shaking flasks was applied to verify the hypothesis that SroLm3 may play a key role in the regulation of secondary metabolism. L33 and L30 were cultured in YEME liquid medium and sampled at 132 h. The metabolic profile was analyzed by the HPLC. Our study revealed that L33 had a more complex metabolic profile in YEME liquid medium in comparison to L30 (Fig. 3a).

We also tried three other liquid media, YMG, MM and R5, and found similar results. But the metabolic profile is not as complex as in YEME liquid medium. This indicates that the promotion of secondary metabolites by in-frame deletion of *sroLm3* is universal in *S. roseosporus*. In addition, the growth rate and biomass production in YEME liquid medium showed no difference between L33 mutant and L30 after exponential growth phase (Fig. S3). That means loss of *sroLm3* had no influence on the cell growth. Therefore, we conclude that deletion of *sroLm3* enhances the expression of genes related to secondary metabolism which results in a higher production of pigment and accumulation of other metabolites.

### 3.3 In-frame deletion of *sroLm3* increases daptomycin and A21978C_1-3_ production in YEME liquid medium

Daptomycin is a cyclic lipopeptide antibiotic produced by *S. roseosporus* L30 [15]. In this study, L33 showed enhanced secondary metabolism in general. Therefore, we performed a shaking flask fermentation experiment using YEME liquid medium and feeding of sodium decanoate to determine if deletion of *sroLm3* can promote the production of daptomycin. Samples from 72 h to 132 h were treated and then analyzed by HPLC.

Data showed that the yield of daptomycin in L33 reached the maximum level at 132 h with an increase of 44.4% in comparison to L30 (Fig. 3b). Additionally, another fermentation experiment revealed three other major derivatives produced by the same gene cluster, A21978C_1-3_, which have a slight difference in their fatty acyl groups [28], reached their top production level during 72–84 h with an improvement by 21.4%∼50.2% in comparison to L30. Since A21978C_1-3_ were the major products of this gene cluster, we presumed the deletion of *sroLm3* can improve the expression of the whole gene cluster, considering the similar growth rate and biomass production of L33.

### 3.4 *sroLm3* is an m4C DNA methyltransferase in *S.roseosporus* L30

**Fig. 4:**
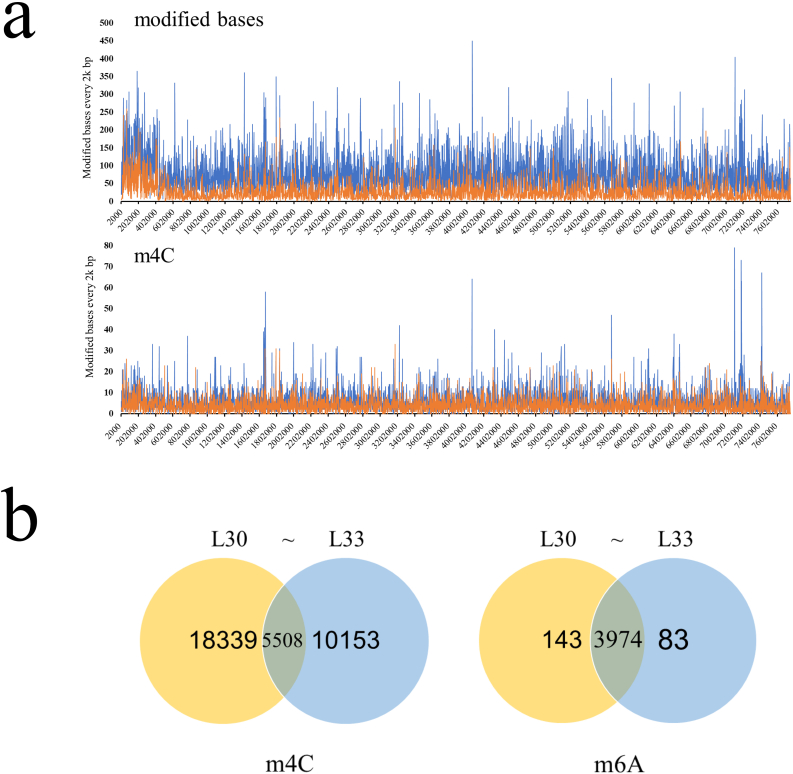
Distribution of modified bases and m4C across the whole genome of L30 and L33 at 72 h. (a) Distribution of disidentified modified bases and detected m4C in the whole genome in L30 and L33 in YEME liquid medium at 72 h. m4C distribution in several BGCs were significantly decreased. (b) Venn diagrams for the distribution of m4C and m6A in L30 and L33 at 72 h. Intersections are the number of modified sites existed in both strains.

To further explore the function of *sroLm3* in the regulation of secondary metabolism, a fermentation experiment with L30 and L33 in YEME liquid medium was performed since this medium is more suitable than the other three media. The 72 h samples were harvested and prepared for SMRT-seq. The methylome data displayed a 34.3% decrease of m4C modification (Fig. 4a, Table S5) but nearly no changes of m6A modification in L33 compared to L30 at 72 h (Fig. 4b). Interestingly, the number of “modified base” in L33 was substantially reduced from L30 maybe due to the difference of the sequencing depth. Most modification on “modified base” happened on guanine but all four kinds of bases can be detected as modified. The conserved motif 5′-CGACNNNCTCC-3’/5′-GGAGNNNGTCG-3′ mentioned above was also fully methylated in the two strains at this time. Interestingly, there were still 5508 remaining m4C modifications (23.1% of the total m4C in L30) and 10153 of the m4C methylations newly appeared in L33 strain. We investigated the bases near the m4C modification and again we found the priority of the methylation occurred on a GCGG motif in both strains. Intriguingly, we found no bias in those two strains and the newfound m4C exhibited no difference compared to L30 strain. (Fig. S4). This data supported the hypothesis that SroLm3 is an m4C methyltransferase but not the only one in *S.roseosporus* L30.

Apart from daptomycin (Cluster 29), *S.roseosporus* is also the producer of some other antibiotics, such as arylomycin (Cluster 10), auroramycin (Cluster 11), napsamycin (Cluster 22) and stenothricin (Cluster 26) [[29], [30], [31], [32]] (Table S6). As mentioned above, m4C modification was unevenly spread across the whole genome but densely distributed in some areas in both strains. Interestingly, up to 8 BGCs revealed decreased m4C distribution compared to L30, especially Cluster 11, 28 and 29 (Fig. S5). This data suggested that loss of SroLm3 induced a global modification change in this region during the stationary stage of the fermentation.

### 3.5 In-frame deletion of *sroLm3* induced a global transcriptional dysregulation in *S.roseosporus* L30

**Fig. 5:**
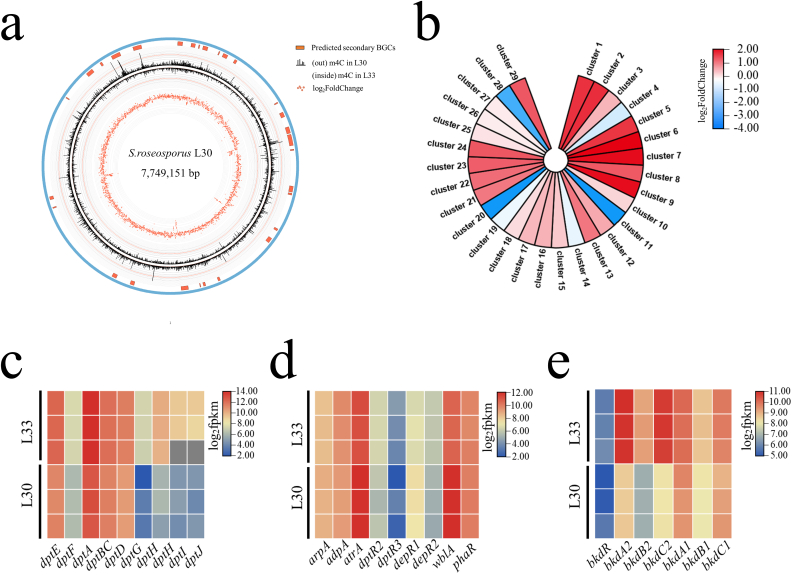
Transcriptome analysis (*n* = 3) between L33 and L30 in YEME liquid medium. (a) The overall correlation between the methylation and transcriptome patterns of L30 and L33. (b) Changes of transcriptional levels (*n* = 3) of core biosynthetic genes of secondary metabolites in L33. Three gene clusters showed significantly decrease of expression. (c) Heatmap of transcriptional levels (*n* = 3) of core genes involved in biosynthesis of daptomycin in L33 compared with L30. All genes were upregulated, and *dptG-J* were significantly upregulated. (d) Heatmap of transcriptional levels (*n* = 3) of regulatory genes of daptomycin biosynthesis in L33 compared with L30. (e) Heatmap of transcriptional levels (*n* = 3) of genes involved in BCDH in L33 compared with L30. *bkdA2B2C2* revealed a significant enhancement.

To further investigate the functions of SroLm3 in *S.roseosporus* L30 and to better understand the mechanisms determining the observed phenotypes, samples (*n* = 3) used for SMRT-seq were also prepared in duplicate for RNA-seq to compare the transcriptomes of L33 and L30 (Fig. 5a). Transcriptome analysis revealed a global alteration occurred in L33 in which there were 1157 upregulated genes that have a notable increase with log2FoldChange≥1 and 736 downregulated genes revealed significant decrease with log2FoldChange ≤ −1 (Fig. S6a). Classification of regulated genes according to their predicted functions in metabolism revealed an enrichment of genes with roles in the biosynthesis of secondary metabolites and metabolic pathways (Fig. S6b). Interestingly, we found 6 regions in which a series of genes showed a decrease (Fig. S6c). Separately, two of them, region 2 and 4, covers Cluster 11 (auroramycin) and Cluster 20 individually; region 1 contains genes predicted as ABC transporter and ribosomal protein and other regions are mostly hypothetical protein.

Totally, up to 7 secondary BGCs displayed significant transcriptional upregulation of core biosynthetic genes, and three of them were significantly downregulated (cluster 11, 20 and 28) with ∣log2FoldChange∣≥ 1 (Fig. 5b). Furthermore, Cluster 11 and 28 also showed a decrease of m4C methylation (Fig. S5). This data supports the hypothesis that SroLm3 dependent m4C methylation has a global regulatory effect on secondary metabolism. Notably, transcriptional levels of core biosynthetic NRPS genes of daptomycin, *dptE-dptD*, and modification genes, *dptG-dptJ* [33], were all upregulated in L33 (Fig. 5c). The enhancement of transcriptional level of genes in the whole BGC was consistent with the improvement of daptomycin's yield in fermentation experiment.

Apart from the core genes, biosynthesis of daptomycin involves many other cellular processes such as amino acid metabolism, fatty acid metabolism and regulatory network (Fig. S7) [34,35]. We collected all the regulators that participate in the regulation of daptomycin biosynthesis based on published literature [[36], [37], [38], [39], [40]] and found nearly no significant changes except for *dptR3*, a MarR family regulator located in BGC of daptomycin [36] and *wblA*, a pleiotropic negative regulator of daptomycin [37] (Fig. 5d). But the gene expression level of *dptR3* is low, and there were no m4C methylated bases in CDS and promoter region of *wblA* in both strains. Furthermore, the downstream regulators of *wblA* revealed either no significant changes (*atrA* and *dptR2*) or low gene expression level (*dptR3*). So, the enhancement in production of daptomycin and its analogs is not directly induced by changes in m4C DNA methylation of known regulator genes.

Daptomycin initiates its biosynthesis with a straight-chain decanoyl group while its analogs, A21978C_1-3_, prefer branched-chain acyl group as their starting unit generated by branched-chain α-keto acid dehydrogenase complex (BCDH complex) [41]. Our previous study revealed that *bkdA1B1C1* is critical for the biosynthesis of A21978C_1-3_, while *bkdA2B2C2* is unnecessary [42]. The transcriptome data showed a notable increase in *bkdA2B2C2* but an unremarkable enhancement of *bkdA1B1C1* and their regulatory gene *bkdR*. This conclusion indicates that the precursor pathway may not contribute to the increase of A21978C_1-3_ production (Fig. 5e).

Transcriptome analysis revealed drastic reductions in gene expression of three BGCs (Cluster 11, 20 and 28). Among those BGCs, Cluster 11 was characterized as the BGC of auroramycin [29] and cluster 28 can produce a secondary metabolite with *m/z* = 405 [32]. Both of them were initially silent BGCs and can be activated by promoter engineering. That means the products of those two BGCs are not among the main secondary metabolites in *S. roseosporus* L30 and will not compete for precursors with biosynthesis of daptomycin. Yet no research identified the products of cluster 20, a type Ⅱ PKS BGC (Fig. S8a). We disrupted the core biosynthetic genes of cluster 20 in WT and L33 and gained two strains without core biosynthetic gene of Cluster 20. Both strains lost the capability to produce red pigment in YEME liquid medium (Fig. S8b) and on R5 solid medium (Fig. S8c). That indicates the Cluster 20 is the BGC of red pigment. We performed a fermentation experiment in YEME liquid medium with two *Δpks20* strains, but no considerable changes in daptomycin production were found (Fig. S8d), as previously shown by transposon mutagenesis [16]. This data indicates that the biosynthesis of red pigment does not compete with daptomycin biosynthetic pathway.

### 3.6 Regulatory genes affected by m4C changes can alter secondary metabolism in *S. roseosporus* L30

As mentioned above, our research revealed that the regulatory network of daptomycin exhibited a dysregulation in L33 strain. *wblA*, a pleiotropic negative regulator [37], exhibited a significant decrease in L33, but the downstream regulators revealed no significant changes or low gene expression level. Interestingly, we counted all regions which contain ≥20 m4C modified sites every 2000 base pairs (≥0.5%) and found that there were 26 transcriptional regulators in total of 87 regions, we believe this is not a coincidence (Fig. S9). Transcriptional regulators take a great part in secondary metabolism [43], so we believe some of these regulators are affected by the changes of m4C distribution in L33. We identified nine regulatory genes with a strong decrease of m4C modification in their coding regions and promoter regions in L33 compared to L30 (Table 3). To identify the function of these regulators in secondary metabolism, we generated a set of mutant strains corresponding to their transcriptional level in L33. In short, upregulated genes were in-frame deleted and downregulated genes were overexpressed in L33.

**Table 3:** Regulatory genes screened out by methylome analysis. Loss of m4C means the number distributed in their coding region.

gene	Predicted function	Loss of m4C^a^	Log_2_FC	m4C in promoter region	padj
*orf1070*	PucR family transcriptional regulator	83.34% (4/24)	−1.03425	0/3	2.07E-31
*orf2061*	transcriptional regulator	100% (0/7)	−0.85267	1/4	9.54E-08
*orf4008*	AraC family transcriptional regulator	87.50% (1/8)	0.109124	0/1	no significance
*orf4141*	Two-component system	75% (2/8)	0.885357	0/0	3.59E-5
*orf4759*	TetR family transcriptional regulator	100% (0/9)	0.383528	0/1	no significance
*orf4820*	IclR family transcriptional regulator	91.67% (1/12)	−0.70305	1/1	3.53E-11
*orf4996*	HxlR family transcriptional regulator	75% (1/4)	−1.30761	0/1	4.50E-7
*orf5274*	Metalloregulator	100% (0/2)	1.692829	0/1	0.047
*orf5980*	Two-component system	62.5% (3/8)	−1.02446	0/0	9.75E-15

a Loss of m4C means the number distributed m4C in their coding region.

**Fig. 6:**
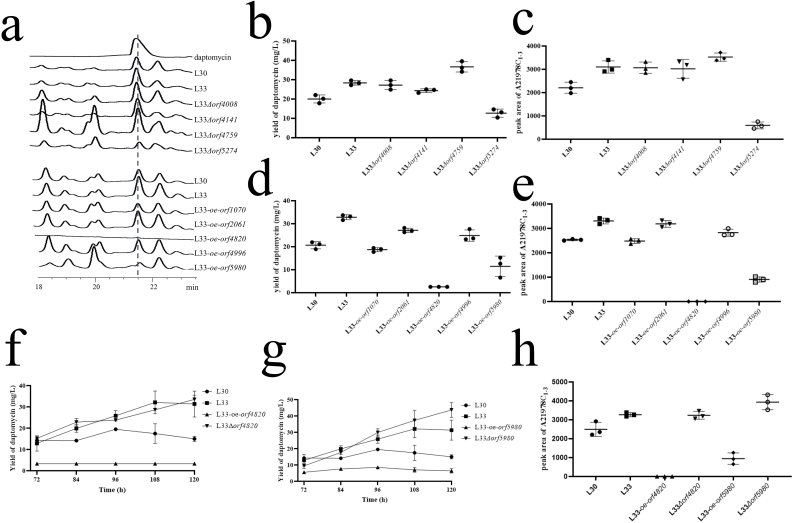
Detection of production of daptomycin by HPLC analysis and morphological changes of the mutants. (a) The peak of daptomycin of L30 and other mutants viewed by HPLC assay. (b–e) Yield of daptomycin and analogs of regulator mutants in YEME liquid medium (*n* = 3, mean with SD). (f–g) Yield of daptomycin in mutant strains of *orf4820* and *orf5980* compared to L30 and L33 (*n* = 3, mean with SD). (h) Yield of analogs of daptomycin in mutant strains of *orf4820* and *orf5980* compared to L30 and L33 (*n* = 3, mean with SD).

All generated strains were identified and prepared for fermentation experiment of daptomycin. The yield of daptomycin was detected by HPLC (Fig. 6a–e). Specially, four mutants revealed significant changes in daptomycin yield. L33*Δorf4759* strain exhibited an enhanced production of daptomycin (+82.9%, +29.1% compared to L30 and L33 separately) while L33*Δorf5274* (−36.4%, −55.1% compared to L30 and L33 separately) and L33-*oe-orf5980* (−44.5%, −65.0% compared to L30 and L33 separately) revealed a significant decrease. Besides, L33*Δorf4759* strain revealed an enhanced production of total analogs (+59.7%, +13.7% compared to L30 and L33 separately) while L33*Δorf5274* (−73.2%, −80.1% compared to L30 and L33 separately) and L33-*oe-orf5980* (−64.3%,-72.7% compared to L30 and L33 separately) revealed a significant decrease.

Additionally, daptomycin, red pigment and other secondary metabolites production was completely disappeared in L33*-oe-orf4820* (Fig. S10a). Besides, L33*-oe-orf4820* strain displayed no aerial hyphae or sporulation when cultured on R5 solid medium (Fig. S10b). Furthermore, some other mutants revealed less production of red pigment, such as L33*Δorf4008*, L33*Δorf4141* and L33*Δorf5274* (Fig. S10a).

We selected two regulators, *orf4820* and *orf5980* for further studies since their influence on daptomycin and analogs production was most significant. Previous studies have shown *orf4820* is an IclR family transcriptional regulator and elucidate its functions in multidrug resistance, inactivation of quorum-sensing signals and sporulation in *Streptomyces coelicolor* [44]. L33-Δ*orf4820* strain exhibited the consistent phenomenon with L33 strain, such as the production of daptomycin (+71.6%, +4.3% compared to L30 and L33 separately) and its analogs (29.8%, 0.01% compared to L30 and L33 separately) (Fig. 6f and h), red pigment, and sporulation (Fig. S10b). Our study revealed its negative regulation of daptomycin and red pigment biosynthesis. EMSA assay verified the direct binding of Orf4820 to the promoter regions of *dptE* and itself (Fig. S10c). The result suggests Orf4820 has a self-regulation and a direct negative regulation in biosynthesis of daptomycin.

*orf5980* is a two-component response regulator of the AmiR/NasT family which responds to a signal cascade and leads to RNA recognition and transcriptional regulation in *Mycobacterium tuberculosis* [45]. In-frame deletion of Δ*orf5980* revealed a 36% enhancement of daptomycin compared to L33, and 124% higher than L30, and a significant enhancement of total analogs (57.5%, 20.1% compared to L30 and L33 separately) (Fig. 6 g-h). Moreover, L33-Δ*orf5980* strain lost the capability of producing red pigment on R5 solid medium (Fig. S10b). This result indicates that Orf5980 is a negative regulator of daptomycin and a positive regulator of red pigment biosynthesis.

Our research verified the hypothesis that regulators affected by m4C DNA methylation could regulate the biosynthesis of secondary metabolites, including daptomycin and red pigment. Considering the wide distribution of regulators in the area which showed less m4C in L33, our study indicates the global regulatory function of m4C DNA methylation.

## 4 Discussion

*Actinomycetes* always show a “metabolic switch” in fermentation experiments during which the cells change from growth phase into stationary phase and activate secondary metabolism [46]. Researchers have described regulatory mechanisms involved in this switch in the past years, including global regulators and environmental factors [47]. The previous studies have uncovered the regulatory mechanism of DNA methylation during phase variation in pathogenic bacteria. But little is known about the relation between epigenetic factors and secondary metabolism in *Actinomycetes*. The application of SMRT sequencing provided a new method to assess the relationship between DNA modification and “metabolic switch” [48]. Our study revealed the existence of two types of DNA modification, m6A and m4C, in *S. roseosporus* L30. m6A is mainly found in a conserved motif 5′-CGACNNNCTCC-3’/5′-GGAGNNNGTCG-3′, while m4C showed a redistribution in the whole genome after the “metabolic switch”. So, we presumed that changes of m4C modification might be associated with the initiation of the secondary metabolism as a part of “metabolic switch”.

In-frame deletion of three candidate DNA methyltransferases confirmed our hypothesis that the loss of DNA MTase will induce morphological changes during the growth. Particularly, SroLm3 was shown to be m4C DNA MTase and is a pleiotropic regulator of secondary metabolites including daptomycin and red pigment according to the morphologic exhibitions and transcriptome analysis. Remarkably, three secondary metabolite gene clusters presented a notable decrease in L33, and two of them, Cluster 11 and Cluster 28 exhibited a decreased m4C amount at the same time. This data verified our hypothesis that SroLm3 could directly or indirectly regulate secondary metabolism.

All genes in the whole BGC of daptomycin were upregulated in L33 compared to L30 but the regulatory genes and precursor-related genes showed no significant changes between L30 and L33. Transcriptome analysis revealed three BGCs with decreased gene expression and two of them were originally silent BGCs. Particularly, one of them, Cluster 20, is responsible for biosynthesis of red pigment and disruption of the core genes of cluster 20 exhibited no significance in production of daptomycin, as was shown previously by transposon mutagenesis [16]. This data suggested that the precursor pathway, the known regulatory network, and red pigment have no impacts on biosynthesis of daptomycin.

**Fig. 7:**
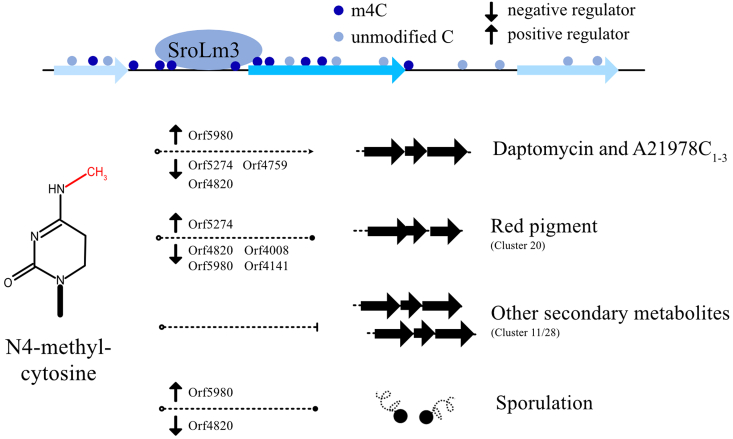
Proposed functional role of SroLm3 in *S.roseosporus* L30.

Analysis in m4C decreasing areas in L33 compared to L30 revealed the preference on secondary metabolism, including secondary BGCs and transcriptional regulators. Among nine regulators located in m4C decreasing areas in L33, four of them exhibited significant effect on production of daptomycin especially *orf4820* and *orf5980*. Orf4820 is a pleiotropic repressor of daptomycin biosynthesis by direct binding with promoter region of *dptE* and *orf4820p*. Orf5980 is negative regulator and we obtained a high-yielding daptomycin producing strain by in-frame deletion. This data indicates that the m4C DNA methylation can indirectly regulate the biosynthesis of secondary metabolites through modifications on pleiotropic regulators (Fig. 7), but the biological mechanism needs to be explored in the future.

## 5 Conclusion

The regulatory mechanism of m6A DNA methylation is documented in bacteria, such as Dam and CcrM. But the impact of m4C modification on secondary metabolism in *Actinomycetes* has not been explored before although m4C is another common DNA modification in bacterial genomes. In this research, we verified that significant loss of m4C can directly or indirectly induce a dysregulation of secondary metabolism in *S. roseosporus*. Since the wide distribution of m4C DNA modification in *Actinomycete*'s genome, our research brings up the potential global regulatory function of m4C in secondary metabolism in *Actinomycetes*.

## Ethics approval

This article does not contain any studies with human participants or experimental animals performed by any of the authors.

## CRediT authorship contribution statement

**Jiao-Le Fang:** Conceptualization, Methodology, Formal analysis, Investigation, Visualization, Writing – original draft. **Wen-Li Gao:** Writing – review & editing, Investigation. **Zhong-Yuan Lyu:** Writing – review & editing, Investigation. **Lie Ma:** Writing – review & editing, Investigation. **Shuai Luo:** Writing – review & editing, Investigation. **Xin-Ai Chen:** Writing – review & editing, Investigation. **Xu-Ming Mao:** Writing – review & editing, Investigation. **Yong-Quan Li:** Funding acquisition, Supervision, Project administration, Writing – review & editing.

## Declaration of interests

All authors declare no competing interests.
