# Supplementary material for: m4C DNA methylation regulates biosynthesis of daptomycin in Streptomyces roseosporus L30
Source: Synth Syst Biotechnol. 2022 Jun 17;7(4):1013–23. doi: 10.1016/j.synbio.2022.06.001 (PMC9240718; doi:10.1016/j.synbio.2022.06.001)
Supplement: Multimedia component 1 [file mmc1.docx]

**Supplementary data**

Table S1: Plasmids used and constructed in this study.

| Plasmids | Description | Reference |
| --- | --- | --- |
| pIJ8661 | Overexpression vector containing a strong promoter *ermEp**, integrative in *Streptomyces* | [19] |
| pKC1139 | Temperature-sensitive shuttle vector for gene knock-out in *Streptomyces* | [20] |
| pTA2 | T vector used for amplification of homologous arms | Toyobo |
| pET28a | Bacterial vector for protein purification | Laboratory |
| pKC1139-*ΔsroLm1* | pKC1139 with homologous arms of *sroLm1* | This study |
| pKC1139-*ΔsroLm2* | pKC1139 with homologous arms of *sroLm2* | This study |
| pKC1139-*ΔsroLm3* | pKC1139 with homologous arms of *sroLm3* | This study |
| pIJ8661-*orf1070* | Overexpression plasmid with *orf1070* | This study |
| pIJ8661-*orf2061* | Overexpression plasmid with *orf2061* | This study |
| pIJ8661-*orf4820* | Overexpression plasmid with *orf4820* | This study |
| pIJ8661-*orf4996* | Overexpression plasmid with *orf4996* | This study |
| pIJ8661-*orf5980* | Overexpression plasmid with *orf5980* | This study |
| pKC1139-*Δpks20* | pKC1139 with homologous arms of core biosynthetic genes of cluster No.20 | This study |
| pKC1139-*Δorf4008* | pKC1139 with homologous arms of *orf4008* | This study |
| pKC1139-*Δorf4141* | pKC1139 with homologous arms of *orf4141* | This study |
| pKC1139-*Δorf4759* | pKC1139 with homologous arms of *orf4759* | This study |
| pKC1139-*Δorf5274* | pKC1139 with homologous arms of *orf5274* | This study |
| pKC1139-*Δorf4820* | pKC1139 with homologous arms of *orf4820* | This study |
| pKC1139-*Δorf5980* | pKC1139 with homologous arms of *orf5980* | This study |
| pET28a-*orf4820* | Protein purification vector for *orf4820* | This study |

Table S2: primers used in this study.

| Primers | Sequence (5’-3’) | Description |
| --- | --- | --- |
| 1 | aagcttCCGTGTAGAGGCGGGACAGT | Construction of pKC1139-*ΔsroLm1* |
| 2 | tctagaGGAGTTCCGGGCCAAGTA |  |
| 3 | tctagaAGTTCCTTCATCGACGTGCTG |  |
| 4 | gaattcCGACCTCGCTGACCTCTATGA |  |
| 5 | tctagaACACGGTCGTCCCCAAGG | Construction of pKC1139-*ΔsroLm2* |
| 6 | ggatccCGGCTGCGGGAAATCG |  |
| 7 | ggatccGGATGTACGACGGACTGACGG |  |
| 8 | gaattcTGGGTGAGACGGGTGGTGT |  |
| 9 | aagcttCGCGGTAGTCGTGCATGTAGT | Construction of pKC1139-*ΔsroLm3* |
| 10 | tctagaTGTCTCGGCAGACGGATCTTTT |  |
| 11 | tctagaGGGCGAGCAAACAACACGG |  |
| 12 | gaattcCGGCACGACCAATTCCGAGA |  |
| 13 | ccaaaggaggcggacatatgGGCTCTCTTCTTCTCAAT | Construction of pIJ8661-*orf1070* |
| 14 | agaagatcgatgtgatatcGAAGATCAGCTCATGCGCAG |  |
| 15 | ccaaaggaggcggacatatgCGCGACCGAGGCCCAC | Construction of pIJ8661-*orf2061* |
| 16 | agaagatcgatgtgatatcCACGACGAACGGGGAACCAC |  |
| 17 | ccaaaggaggcggacatatgACAGCGGAGACTTCCCAGAC | Construction of pIJ8661-*orf4820* |
| 18 | AgaagatcgatgtgatatcAGAGCACGCACCCAATCTAACC |  |
| 19 | ccaaaggaggcggacatatgGAAGAAGGCACTTCGAAGT | Construction of pIJ8661-*orf4996* |
| 20 | agaagatcgatgtgatatcGCGTTCTCACGGTCGTCTTCG |  |
| 21 | ccaaaggaggcggacatatgACCACCCCCGAGTCGC | Construction of pIJ8661-*orf5980* |
| 22 | agaagatcgatgtgatatcTGCCTACGTATGCGTGGTGT |  |
| 23 | gggctgcaggtcgactctagaGCCTTGCTTCAGCGACTTACTT | Construction of pKC1139-*Δorf4008* |
| 24 | tccaccgtcccgaaccCGCCCTCGAAGAGCACGAT |  |
| 25 | GGTTCGGGACGGTGGACA |  |
| 26 | tgattacgaattcgatatcCGACCCAAGGGAACCTGTGAA |  |
| 27 | gggctgcaggtcgactctagaGGCGTCAACTGCTGTGGCT | Construction of pKC1139-*Δorf4141* |
| 28 | ggaggaccccacatgaGGTGTCGGTGGCGAAGGAA |  |
| 29 | TCATGTGGGGTCCTCCGTGGTG |  |
| 30 | ctatgacatgattacgaattcCGGGCGTTTCGCTCTGGTAT |  |
| 31 | gggctgcaggtcgactctagaGCCCTTCTCGTTGATGCCG | Construction of pKC1139-*Δorf4759* |
| 32 | agccgccagtacagcgGGTTCCCCGTTCGGTGGT |  |
| 33 | CGCTGTACTGGCGGCTCGTC |  |
| 34 | tgattacgaattcgatatcGCGGCTTCTGCCTGGACTT |  |
| 35 | gggctgcaggtcgactctagaCCCTGTGACCTGTACGAACC | Construction of pKC1139-*Δorf5274* |
| 36 | ccttcacttcggcaggTGCTCCGCAACACCAAGTG |  |
| 37 | CCTGCCGAAGTGAAGGTTGA |  |
| 38 | tgattacgaattcgatatcATCCCGTACCCGCTCAGATA |  |
| 39 | GGCTGCAGGTCGACtctagaGTCCTCGCTCCGCTATCTGCTC | Construction of pKC1139-*Δpks20* |
| 40 | CACGCATCGCTGTCCACCCT |  |
| 41 | GGACAGCGATGCGTGTCACCGTCCTCGATGAGCCG |  |
| 42 | TATGACATGATTACgatatcCCACCTTCCGGCTCACCAT |  |
| 43 | GGGCTGCAGGTCGACTCTAGAGGCCGCGTCCAAGAAGATC | Construction of pKC1139-*Δorf4820* |
| 44 | tgacgacaccgacgctACAGCTCGGTCACGGTCAGG |  |
| 45 | AGCGTCGGTGTCGTCATGC |  |
| 46 | TGATTACGAATTCGATATCAGGGTTACGCCCACTTGCTC |  |
| 47 | GGGCTGCAGGTCGACTCTAGAGTAGGGCGGCTCAGTATTTCG | Construction of pKC1139-*Δorf5980* |
| 48 | ttcttctcctcggcgtCCTCCTCCTCCAGCATCTCC |  |
| 49 | ACGCCGAGGAGAAGAAGAAGT |  |
| 50 | TGATTACGAATTCGATATCCGAGAACAAGGCCGAACACC |  |
| 51 | TAAGAAGGAGATATACCATGACAGCGGAGACTTCCCAGAC | Construction of pET28a-*orf4820* |
| 52 | TTGTCGACGGAGCTCGAATTccgcagcgcgtccgccac |  |

Table S3 Methods used in HPLC assay.

A: detection of metabolic profile:

| Mobile phase | A | ddH_2_O |
| --- | --- | --- |
|  | B | acetonitrile with 0.1% (v/v) formic acid |
| Column | Agilent Extend XDB-C18 150/4.6mm | |
| Flow Rate | 1.0 mL/min | |
| Wavelength | 190-600 nm | |
| Column Temp | 30 °C | |
| Injection Volume | 50 μL | |
| Gradient | time (min) | mobile phase (v/v) |
|  | 0 | 85:15 |
|  | 2 | 85:15 |
|  | 22 | 42:58 |
|  | 24 | 0:100 |
|  | 27 | 0:100 |
|  | 27.5 | 85:15 |
|  | 30 | 85:15 |

B detection of daptomycin

| Mobile phase | A | ddH_2_O |
| --- | --- | --- |
|  | B | acetonitrile with 0.1% (v/v) trifluoroacetic acid (TFA) |
| Column | Agilent Zorbax 300SB-C18 250/4.6mm | |
| Flow Rate | 1.0 mL/min | |
| Wavelength | 215 nm | |
| Column Temp | 30 °C | |
| Injection Volume | 50 μL | |
| Gradient | time (min) | mobile phase (v/v) |
|  | 0 | 90:10 |
|  | 5 | 65:35 |
|  | 55 | 45:55 |
|  | 60 | 5:95 |
|  | 63 | 90:10 |
|  | 65 | 90:10 |

Table S4: SMRT sequencing of *S. roseosporus* L30 exhibits two types of DNA methylation

| **Time**  **Type** | **24h** | **72h** |
| --- | --- | --- |
| m4C | 6279 | 6788 |
| m6A | 4299 | 4415 |
| Modified base* | 84475 | 69394 |
| total | 95053 | 80597 |

***** Bases with unidentified modification.

Table S5: Modified bases detected by SMRT sequencing in 72 h samples of L30 and L33

| Strain  Type | L30 | L33 |
| --- | --- | --- |
| m4C | 23,847 | 15,661 |
| m6A | 4,117 | 4,057 |
| modified base* | 268,805 | 87,441 |
| total | 296,769 | 107,159 |

* Bases with unidentified modification.

Table S6: Predicted gene clusters of secondary metabolites accessed by antiSMASH 6.0 database.

| Cluster | Type | From | To | Most similar known cluster | Similarity | Observed compounds |
| --- | --- | --- | --- | --- | --- | --- |
| Cluster 1 | NRPS, T1PKS | 74340 | 123667 | kanamycin | 2% |  |
| Cluster 2 | T3PKS | 210686 | 251738 | tetronasin | 11% |  |
| Cluster 3 | melanin | 286629 | 297111 | melanin | 100% |  |
| Cluster 4 | NRPS, T1PKS | 331117 | 379959 | valinomycin / montanastatin | 13% |  |
| Cluster 5 | RiPP-like | 496550 | 506294 | tetronasin | 3% |  |
| Cluster 6 | NRPS, T1PKS | 545026 | 593221 | SGR PTMs | 100% | photocyclized alteramide A [32] |
| Cluster 7 | terpene | 673151 | 699275 | hopene | 69% |  |
| Cluster 8 | RiPP-like | 1273758 | 1285110 |  |  |  |
| Cluster 9 | NRPS,T1PKS | 1385035 | 1434561 | collismycin A | 74% |  |
| Cluster 10 | NRPS | 1541265 | 1606708 | arylomycin | 100% | arylomycin [30] |
| Cluster 11 | oligosaccharide, T1PKS | 1619135 | 1731106 | auroramycin | 79% | auroramycin [29] |
| Cluster 12 | siderophore | 1768576 | 1781664 |  | 3% |  |
| Cluster 13 | thioamide-NRP | 2139524 | 2198871 | BD-12 | 17% |  |
| Cluster 14 | terpene | 2209999 | 2227952 |  |  |  |
| Cluster 15 | lanthipeptide-class-iii | 2590428 | 2611417 | AmfS | 100% |  |
| Cluster 16 | T1PKS, NRPS-like | 2701928 | 2754053 | enduracidin | 10% |  |
| Cluster 17 | lanthipeptide-class-ii | 3363380 | 3385708 | SRO15-3108 | 100% |  |
| Cluster 18 | lassopeptide | 3518320 | 3541116 | keywimysin | 100% |  |
| Cluster 19 | siderophore | 3567820 | 3575753 | desferrioxamin B | 60% |  |
| Cluster 20 | T2PKS | 3693925 | 3766455 | benastatin A-B | 33% | Red pigment |
| Cluster 21 | phosphonate, betalactone | 4240569 | 4281399 | FR-900098 | 100% | FR-900098 [32] |
| Cluster 22 | NRPS-like | 4430889 | 4477032 | napsamycin A-D / mureidomycin A-B | 89% | napsamycin [31] |
| Cluster 23 | siderophore | 5384528 | 5396306 | desferrioxamin B | 100% |  |
| Cluster 24 | lanthipeptide-iii / ii | 5474266 | 5505387 |  |  |  |
| Cluster 25 | ectoine | 6525638 | 6536036 | ectoine | 100% |  |
| Cluster 26 | NRPS, NRPS-like | 6838059 | 6911432 | stenothricin | 95% | stenothricin [31] |
| Cluster 27 | terpene | 7009517 | 7030593 | steffimycin D | 19% |  |
| Cluster 28 | T1PKS, NRPS-like, nucleoside | 7140346 | 7232699 | divergolide A-D | 44% | m/z 405 [32] |
| Cluster 29 | NRPS | 7372750 | 7457771 | daptomycin | 100% | daptomycin [15] |

Figure S1: Modified bases existed in genomes of *S.roseosporus* L30 detected by SMRT-seq. (a) Distribution of modified bases in the whole genomes during the time of 24h and 72h. (b) distribution of m6A and m4C DNA methylation in the whole genomes at the time point 24h and 72h. (c) Venn diagram of m4C and m6A DNA methylation in *S.roseosporus* L30 at the time point 24h and 72h. (d) Frequency of base occurrence around m4C in samples of 24h and 72h.


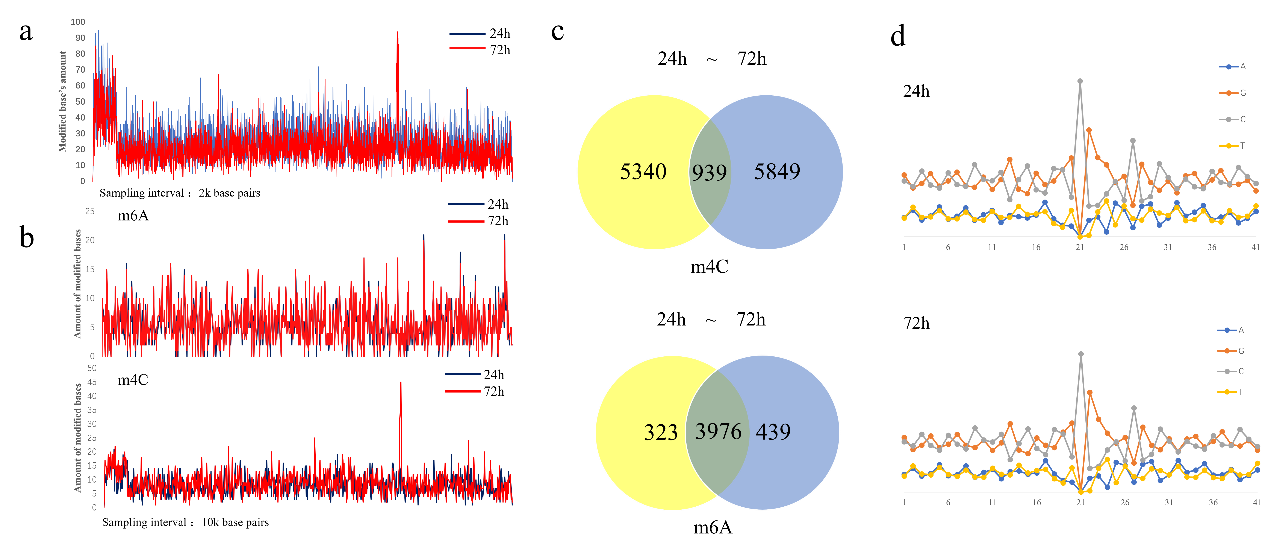


Figure S2: Schedule of in-frame deletion and identification of in-frame deletion strain of three candidates. (a) Schedule of in-frame deletion of three candidates in *S.roseosporus* L30. Arrow pairs marks primer pairs used for identification of the mutants. (b-d) Results of the identifications of three mutants using PCR.


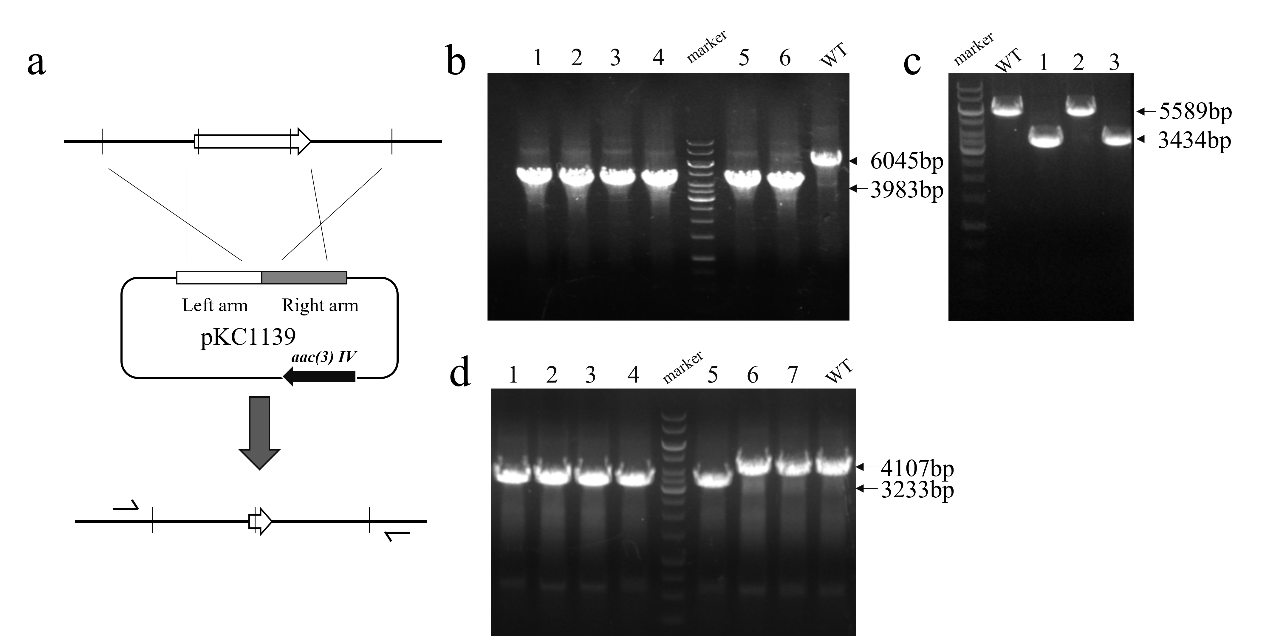


Figure S3: Growth curve of L33 and L30 in YEME liquid medium. Fermentation experiment is applied in YEME liquid medium. Samples (*n*=3) were centrifuged, and supernatant was removed as much as possible. Precipitates were placed in stove with a temperature of 55℃ for 36h and accurately weighed using BT125D electronic balance (d=0.01mg, Sartorius). Red pigment accumulated rapidly after 60h,so the dry weight is not precise in later phase.


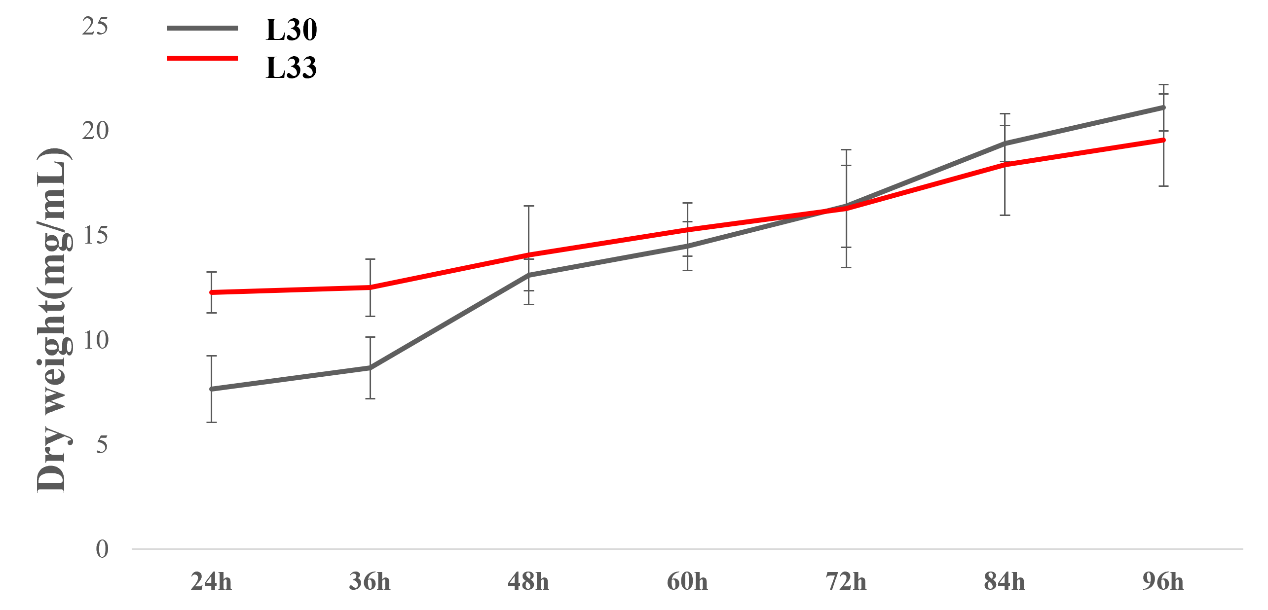


Figure S4: Frequency of base occurrence around m4C in 72h-samples of L30 and L33.


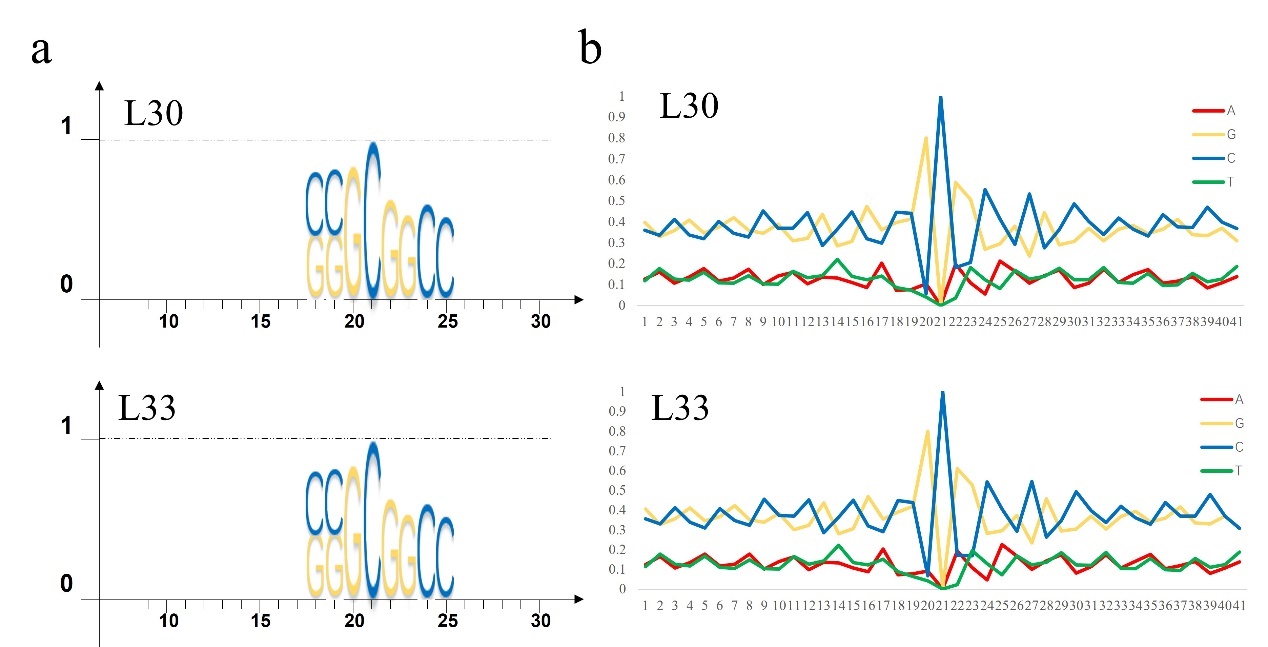


Figure S5: The Distribution of m4C DNA methylation in the whole genome (a) and BGC of Cluster 11, 28 and 29 (b).


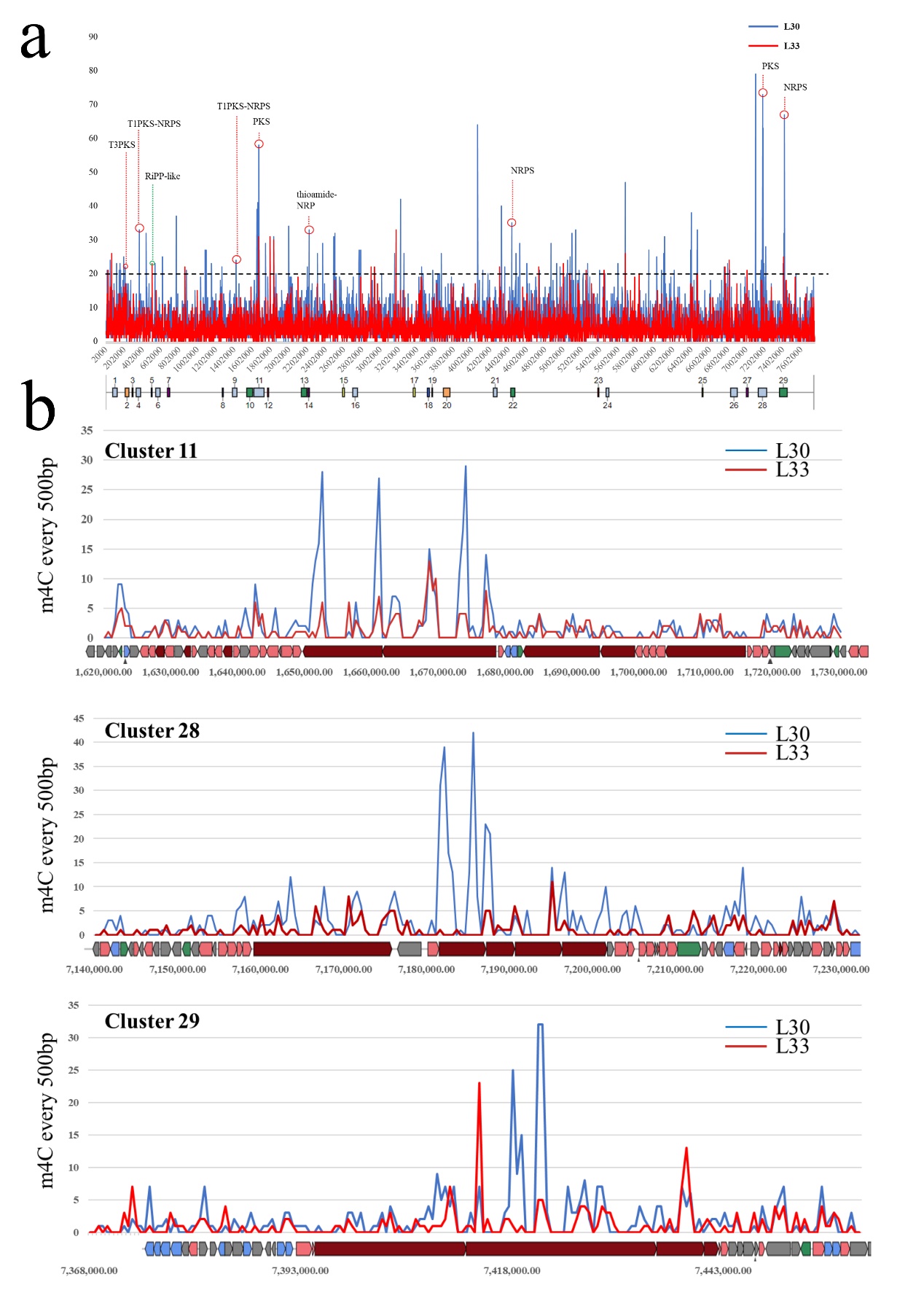


Figure S6: Overview of the transcriptome analysis. (a) Volcano plot of regulated genes in L33 compared with L30. (b) Classification of regulated genes according to their predicted functions. The number of genes that are differentially expressed are grouped based on KEGG (Kyoto Encyclopedia of Genes and Genomes). (c) 6 regions with clustered decrease in gene expression.


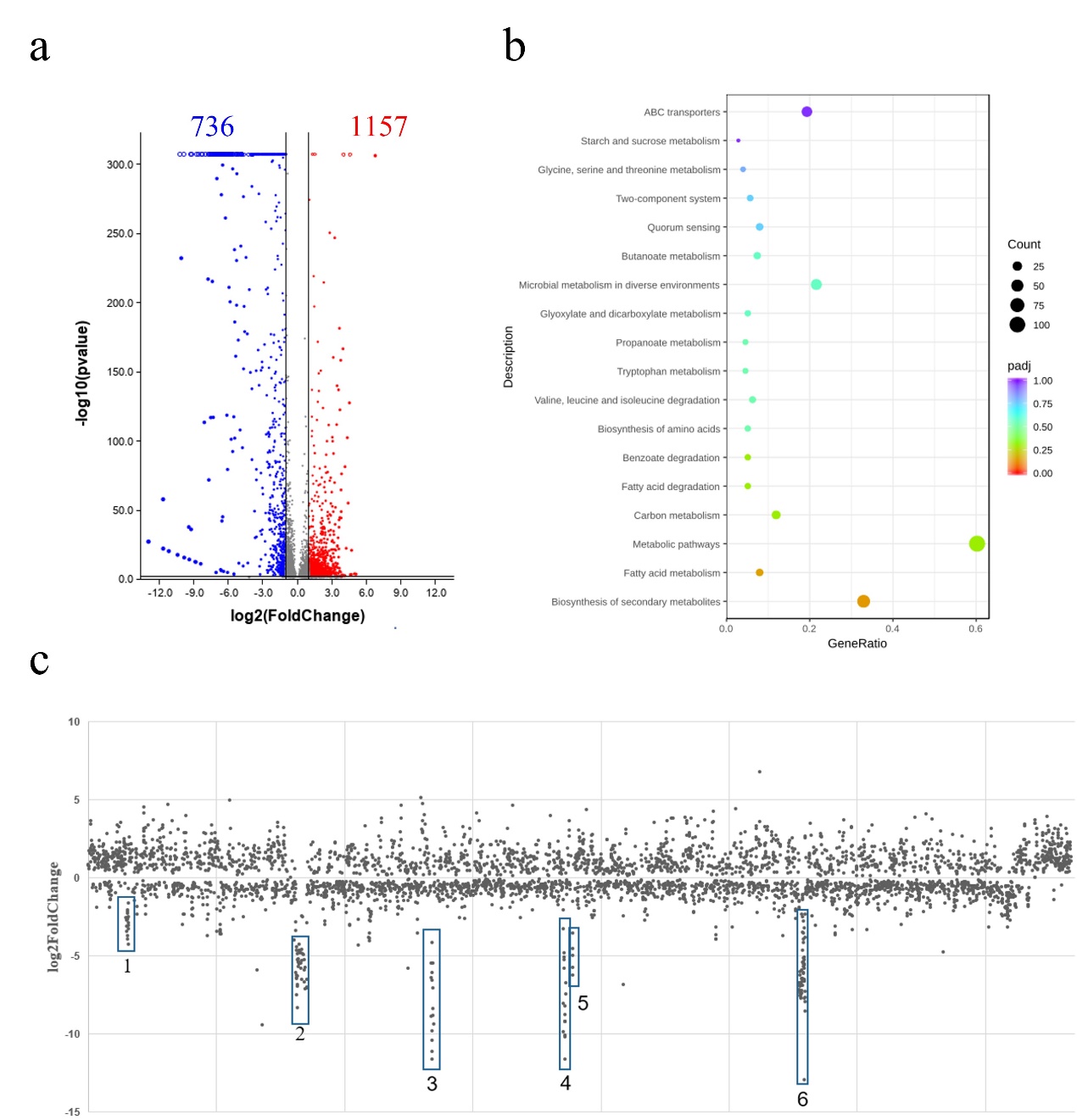


Figure S7: An overview of the network of daptomycin biosynthesis (some regulators showed in the figure were not analyzed in Figure 5d since we found no available data about those regulators ,such as BldD).


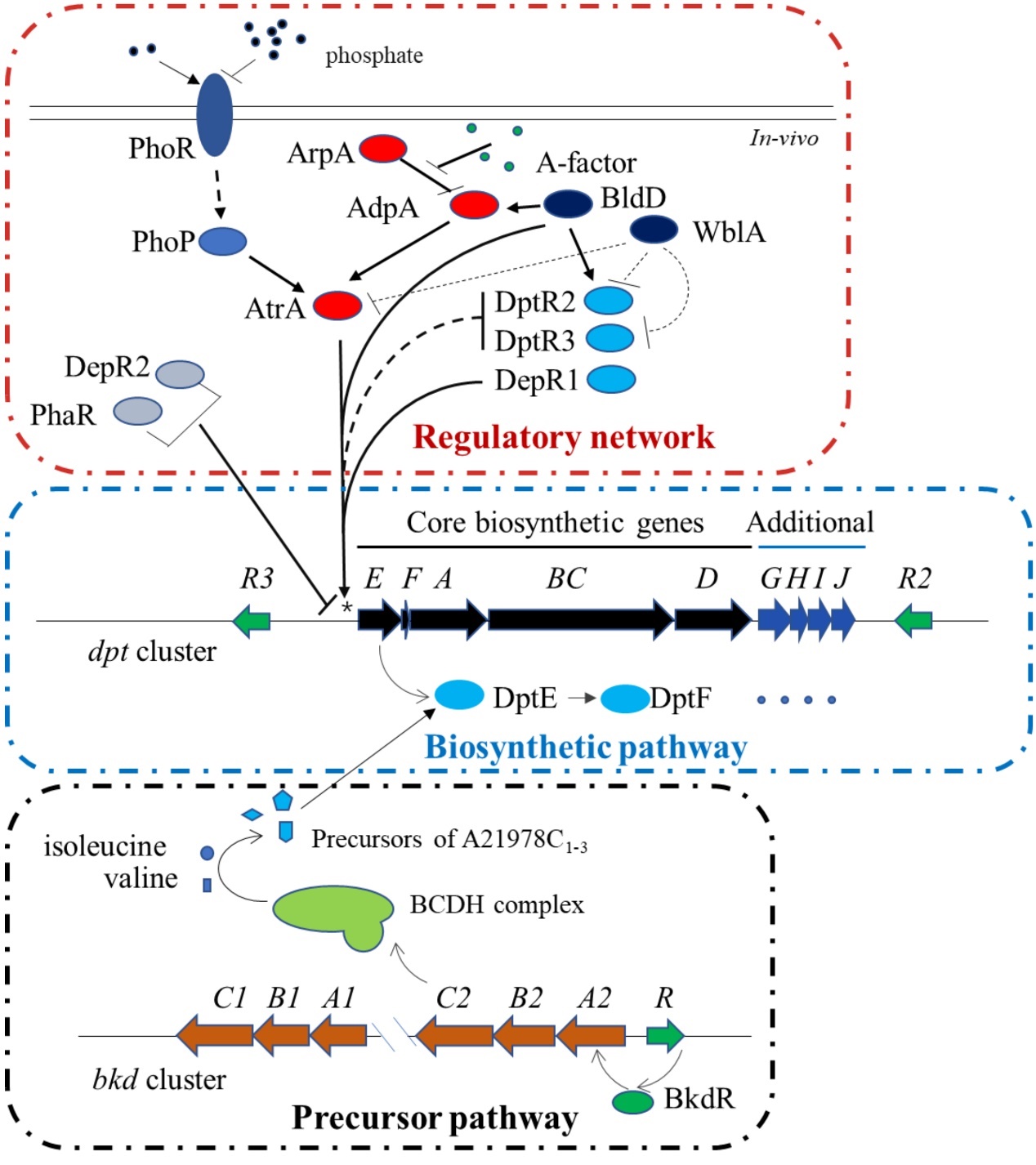


Figure S8: Overview of the cluster 20 and the morphologic changes of in-frame deletion strains (a) Overview of the cluster 20. (b) morphologic changes of in-frame deletion strains in YEME liquid medium. (c) Morphologic changes of in-frame deletion strains on R5 solid medium. (d) Daptomycin production of in-frame deletion strains compared to L30 and L33 (*n*=3, mean with SD ).


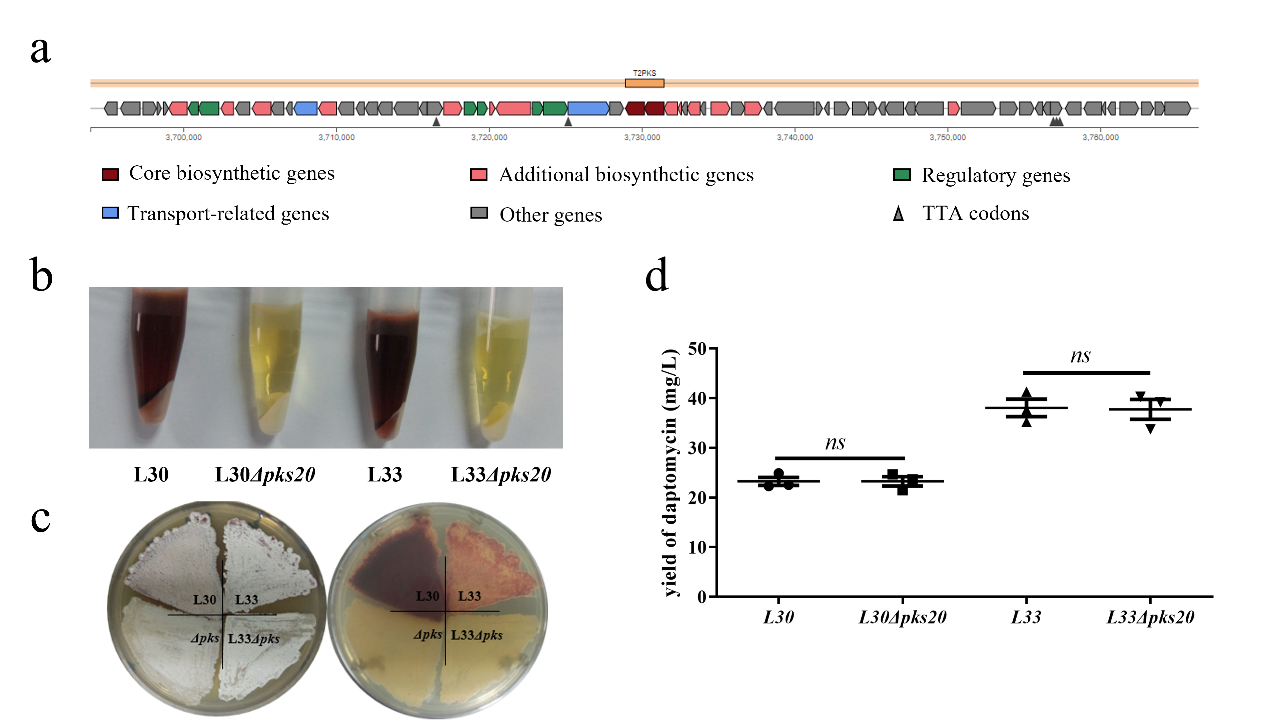


Figure S9: Predicted functions of genes located in regions with ≥20 m4C every 2,000 base pairs.

Figure S10: (a) Morphological diversities of mutant strains of regulators in YEME liquid medium. (b) Morphological diversities of mutant strains of regulators of orf4820 and orf5980 on R5 solid medium. (c) EMSA assay to verify the binding with promoters of *orf4820* and *dptE*.

**
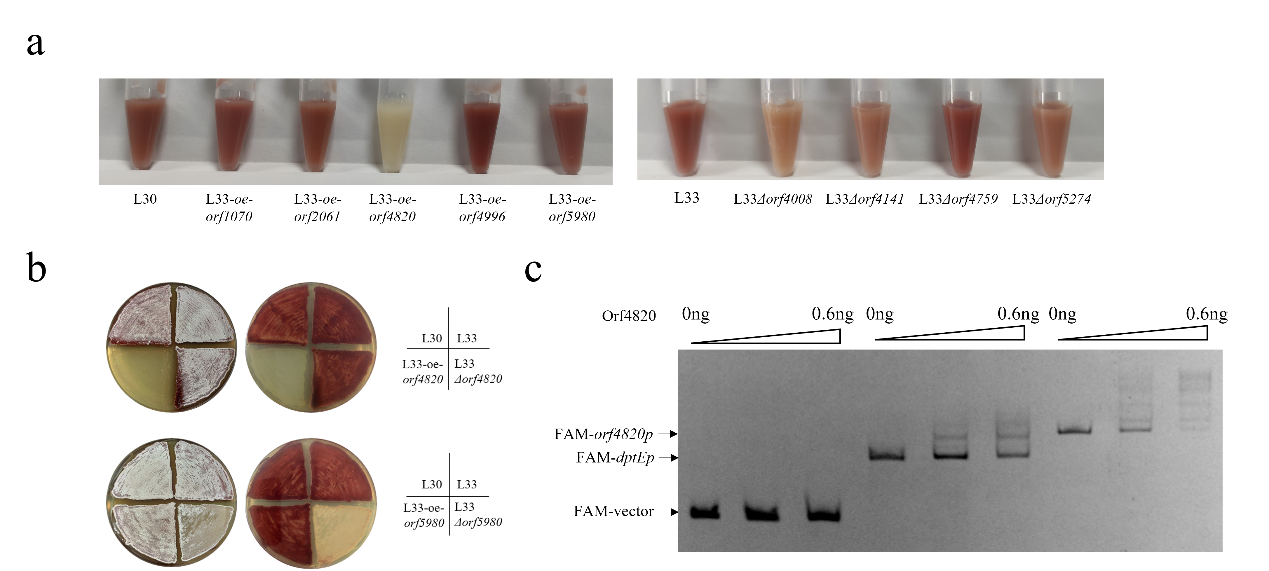
**
